# Nasally-Administered Oxytocin Has Limited Effects on Owner-Directed Attachment Behavior in Pet Dogs *(Canis lupus familiaris)*

**DOI:** 10.3389/fpsyg.2017.01699

**Published:** 2017-09-29

**Authors:** Lauren E. Thielke, Giovanna Rosenlicht, Sarina R. Saturn, Monique A. R. Udell

**Affiliations:** ^1^Human-Animal Interaction Lab, Animal Rangeland Sciences, Oregon State University, Corvallis, OR, United States; ^2^Department of Psychological Sciences, University of Portland, Portland, OR, United States

**Keywords:** oxytocin, secure base, dogs, social behavior, human-animal interactions, attachment style

## Abstract

The present study explored the effects of intranasal oxytocin, a naturally occurring hormone, on the behavior of pet dogs during an attachment test. Each dog participated in two testing sessions. On one visit saline was administered nasally, and on another, oxytocin was administered nasally. For half of the dogs (*n* = 20), solutions were administered with a Mucosal Atomization Device (MAD) and for half of the dogs (*n* = 20), solutions were administered using a nasal spray bottle. Condition order was counterbalanced and a double-blind methodology was employed. Following a 30-min wait period after administration of solutions, dog-owner pairs participated in the Secure Base Test, a short attachment test consisting of three 2-min phases: (1) Baseline- the owner was present, dogs were able to freely explore the testing room (2) Alone- dogs were left alone in the testing room (3) Return- owners re-entered the room and were reunited with their dog. In each phase the dog was evaluated for contact seeking, exploration, and avoidance behaviors. Although, oxytocin administration was expected to increase owner-directed proximity and contact seeking behavior, this effect was not observed. In fact, in the baseline phase, dogs spent significantly more time seeking the proximity of their owners when they received saline than when they received OT (*p* < 0.05). Sex differences were also assessed for the behavioral variables of interest in the Secure Base Test, and results indicated that OT did not affect dogs' behavior in the alone phase, but when saline was administered, females spent significantly more time in contact with the door than males in the alone phase (*p* < 0.05). Overall, the effects of nasally administered oxytocin on attachment related behavior appeared to be limited or inconsistent for this pet dog population.

## Introduction

Oxytocin is a hormone involved in the formation and maintenance of social bonds between a variety of mammalian species (for a review, see Carter et al., 2008), including dogs and humans (for a review, see Beetz et al., 2012). Dogs have been found to display increased OT levels within a variety of social contexts involving exposure to a familiar person after a separation including when the human engages in verbal praise and physical contact with the dog, verbal praise alone, or even when the dog simply has visual access to the person (Rehn et al., 2014). Humans may also experience elevated OT after interacting with their dogs, however results have been mixed or gender dependent. For example, Miller et al. (2009) demonstrated that OT levels increase in women after interaction with a bonded dog, but this effect was not seen in men. Evidence of a correlation in OT levels between dogs and their female owners has also been found (Handlin et al., 2011, 2012). Furthermore, there is evidence that owners who report having positive relationships with their dogs have higher urinary OT concentrations after interacting with their dogs, and in turn, their dogs gaze at them for longer durations in comparison to owners who report having less positive relationships with their dogs (Nagasawa et al., 2009).

Given OT's important role in relationships between dogs and humans, a growing area of research has explored the effects of nasally administered OT on dogs' behavior in a variety of contexts. In one study, dogs displayed more affiliative behaviors toward familiar humans and conspecifics when given OT nasally, compared to when they received saline (Romero et al., 2014). Another study demonstrated that female dogs gazed at their owners more after OT was administered intranasally, and urinary OT concentrations of their owners increased as a result, but these effects were not seen in male dogs and their owners (Nagasawa et al., 2015). Other studies have shown that administering OT nasally to dogs influences performance on object-choice tasks (Oliva et al., 2015), influences play behavior between familiar conspecifics (Romero et al., 2015), induces positive expectancy biases (Kis et al., 2015), and modulates reactions to owners and strangers in a threatening approach test (Hernádi et al., 2015). In addition, OT has recently been shown to have an impact on dogs' motion perception in a study comparing dogs' responses to a two-dimensional projection of a moving human (a “biological stimulus”) and to the same video when scrambled and inverted, after OT or saline administration (Kovács et al., 2016). Dogs that received OT spent less time looking at the biological stimulus than dogs receiving saline indicating that dogs may have a natural preference for the motion of biologically-relevant stimuli, and OT administration decreases this preference.

While oxytocin is often associated with bond formation, to date, no studies have directly investigated whether increased OT levels might influence the secure base effect, or other behaviors indicative of “secure” attachments of pet dogs toward their owners. One of the first investigations into dog-human attachment utilized a seven episode version of the Strange Situation Test, originally designed to evaluate a human infant's attachment to their mother (Topál et al., 1998). Since then a variety of approaches to the study of dog-human attachment behavior- including the use of methodologies with additional controls and counterbalanced conditions- have been used successfully (Palmer and Custance, 2008; Rehn et al., 2013). The majority of these studies would suggest that dogs are capable of forming attachment bonds to their human caregivers, which includes proximity seeking behavior and preference for their owner. However, another important component of attachment is the secure base effect. Beyond the basic attachment bond, the secure base effect requires that a bonded individual be able to use their attachment figure as a source of comfort when challenged or stressed (Bowlby, 1969). Currently it is unknown whether OT might (1) exclusively promote increased proximity seeking and gaze behavior toward a human attachment figure (Romero et al., 2014; Nagasawa et al., 2015) independent of attachment security or (2) whether OT administration also facilitates feelings of security (the secure base effect). This distinction would have important implications for understanding the role of OT in bond formation and maintenance, and could also have important applied implications for the treatment of social anxiety disorders (see Thielke and Udell, 2015 for a review).

There is also a great need for additional double blind replications of studies measuring the behavioral outcomes of intranasal oxytocin administration in dogs in general. Relatively few studies have been conducted that directly evaluate how this procedure influences the behavior of dogs toward their owners, and of those that do exist, the effects have often been relatively small (Hernádi et al., 2015; Oliva et al., 2015; Romero et al., 2015).

Therefore, the current study asked how administration of intranasal oxytocin would affect the attachment behavior, and attachment style, of dogs in both the presence and absence of their owners. Given that we were specifically interested in the secure base effect, we utilized a modified version of the test originally developed by Harlow (1958) designed to study the secure base effect in infant rhesus macaques *(Macaca mulatta)*. This Secure Base Test consisted of three episodes, each 2 min in length: Baseline- dogs experienced an unfamiliar testing room in the presence of their owners, Alone- a phase in which owners left dogs in the room alone, and Reunion- in which the owner returns. As in expanded version of this test, including the SST, the return phase is critical for assessing greeting and proximity seeking behavior, as well as assessing the style of attachment to the owner (Waters, 1978; Rehn et al., 2013). Importantly a double-blind methodology was used to prevent expectations of the OT administrator, experimenter, owners or coders from influencing the behavior of the dog. If OT administration had a significant influence on the attachment behavior of dogs toward humans, we predicted that it might function in one of two ways:

1. If OT increases affiliative behavior in dogs, time spent in contact with, and in proximity to, owners should increase when dogs receive OT vs. saline. In the absence of a secure attachment, increased motivation to seek the proximity of the owner could result in increased rates of search and anxiety related behaviors when the dog is left alone.
2. If OT increases feelings of security in attachment relationships, then dogs should seek the proximity of their owner but also (a) display fewer stress-related behaviors when left alone after receiving OT and (b) be more likely to exhibit behaviors associated with a secure attachment after OT administration (reunion behavior, contact-exploration balance) when compared to saline administration.

Additionally, we thought it was possible, that if OT increases affiliative behavior in dogs, OT could also increase attachment security or result in changes in attachment style. Although it may take several interactions between individuals for an attachment relationship to form, dogs living in shelters have been shown to form this relationships relatively quickly-after just a few interactions with an experimenter (Gàcsi et al., 2001). Furthermore, research with humans has shown that a significant proportion of men who were classified as insecurely attached prior to a single dose of OT administration displayed increased attachment security on an attachment task (Buchheim et al., 2009).

A secondary aim of this study was to test two administration methods-a nasal spray bottle and a Mucosal Atomization Device (MAD) to determine if the methods differ in the amount of stress-related behaviors that dogs display upon administration. One study used a nasal spray bottle (Romero et al., 2014) while another study used MADs for administration (Oliva et al., 2015). Thus, we aimed to evaluate whether stress responses during administration differed depending on the type of administration device used, as differences in stress during administration could impact results of behavioral tests.

## Materials and methods

### Subjects

A total of 40 pet dogs were volunteered by their owners. All dogs were required to be over 10 months of age, in good health, not have a history of separation anxiety and not be pregnant or lactating, due to oxytocin's known role in inducing labor and lactation. As breed was not a variable under evaluation, a variety of breeds and mixes were enrolled in the study. There were 18 male and 22 female subjects. Table 1 lists each dog's breed and age.

**Table 1:** Breed, age, treatment order, and attachment style categorizations for each subject.

**Dog's Name**	**Age (years)**	**Breed**	**Treatment Order**	**OT Attachment Style**	**Saline Attachment Style**
Annie	5	American Pit Bull Terrier Mix	OT-Saline	Secure	Secure
Annie	10	Border Collie	OT-Saline	Secure	Unclassifible
Blue	2	Dachshund	Saline-OT	Secure	Secure
Bohdie	3	American Pit Bull Terrier Mix	Saline-OT	Secure	Secure
Boss	7.5	German Shepherd	OT-Saline	Insecure avoidant	Insecure disorganized
Bree	4.7	Collie	Saline-OT	Insecure disorganized	Unclassifible
Bruno	5.3	Poodle/Border Collie/Papillion Mix	Saline-OT	Secure	Secure
Carmella	9.6	Golden Retriever	OT-Saline	Insecure ambivalent	Insecure ambivalent
Ducky	3.5	Corgi	OT-Saline	Insecure avoidant	Secure
Ellie	3.9	Cane Corso	Saline-OT	secure	Secure
Ember	3	Border Collie	Saline-OT	Insecure ambivalent	Insecure ambivalent
Grace	10	Golden Retriever	Saline-OT	Secure	Secure
Gryphon	8	Black Russian Terrier	OT-Saline	Insecure avoidant	Insecure avoidant
Guinness	4	Standard Poodle	OT-Saline	Secure	Secure
Hampton	6.7	Rottweiler/American Pit Bull Terrier Mix	OT-Saline	Secure	Secure
Honey	2	Labrador Retriever Mix	Saline-OT	Secure	Secure
Ian	8	Border Collie	OT-Saline	Secure	Secure
Jac	1.5	Brittany Spaniel	Saline-OT	Secure	Secure
Jade	1.75	Black Russian Terrier	OT-Saline	Secure	Secure
Kenny	3.2	Golden Retriever	Saline-OT	Secure	Secure
Kobe	7	Akita/American Pit Bull Terrier Mix	Saline-OT	Secure	Secure
Lily	5.3	Border Collie Mix	Saline-OT	Secure	Secure
Lizzie	8	Australian Shepherd Mix	Saline-OT	Insecure ambivalent	Insecure avoidant
Loke	3	Alaskan Malamute	Saline-OT	Secure	Secure
Louie	2.6	Labrador Retriever Mix	OT-Saline	Secure	Secure
Molly	2.2	Shepherd/Husky/Labrador Retriever/American Pit Bull Terrier Mix	Saline-OT	Secure	Secure
Pumpkin	3	Australian Cattle Dog Mix	Saline-OT	Secure	Secure
Raven	1	Dachshund	OT-Saline	Secure	Secure
Riley	8	Golden Retriever	OT-Saline	Secure	Secure
Ripley	1	Border Collie	OT-Saline	Secure	Secure
Rowan	10 months	Australian Shepherd	Saline-OT	Secure	Secure
Shelby	11	Golden Retriever	Saline-OT	Secure	Secure
Tahoma	3	Labrador Retriever	OT-Saline	Secure	Secure
Tara	11	American Pit Bull Terrier Mix	OT-Saline	Secure	Secure
Teddy	13.5	Shetland Sheepdog	OT-Saline	Insecure avoidant	Unclassifible
Tenaya	12.9	Collie	OT-Saline	Secure	Secure
Willow	12.5	Collie	OT-Saline	Secure	Secure
Wrigley	7	Labrador Retriever/Akita Mix	Saline-OT	Secure	Secure
Zoey	4	Australian Shepherd/McNab Shepherd/Border Collie Mix	OT-Saline	Secure	Secure
Zum	11	American Pit Bull Terrier Mix	Saline-OT	Secure	Secure

### Ethics approval statement

This study was conducted under ethical approval from the Institutional Animal Care and Use Committee (IACUC) of Oregon State University (ACUP number 4664). Consent was provided by dog owners via a consent form approved by Oregon State University's IACUC committee.

### Experimental protocol

The order of treatments in this study (saline vs. OT) was counterbalanced. Fifty microgram (24 IU) of OT (Extreme Peptide, United States) were diluted in 0.5 ml of a 0.65% saline solution (Ocean Saline Nasal Spray, Bridgewater, NJ). Dogs receiving the saline solution received 0.5 ml of the 0.65% saline solution. All solutions were prepared within 48 h of each testing session and given a code associated with the testing session by an assistant who did not participate in testing. This kept the experimenter blind to the solution being administered during the test. OT or saline was administered intranasally in an unfamiliar room by an experimenter.

Nasal administration methods have varied across scientific studies. Therefore, in the current study we utilized and compared two previously cited administration methods: A nasal spray bottle (Romero et al., 2014) and a MAD (Oliva et al., 2015). Half of the subjects (*n* = 20) experienced the nasal spray bottle (Sinox Pharma, China) administration method and the other half experienced the MAD (Live Action Safety, Eugene, Oregon) administration method for both their OT and saline administrations. For each type of administration, food was placed in a container so that the dogs could smell it but not access it in order to ensure that they were sniffing while the administration occurred. Each dog participated twice on 2 different days (visits were spaced at least 5 days apart) receiving either saline or OT prior to the attachment test. Administration was filmed and videos were coded for stress-related behaviors (Table 2). Thirty percent of the videos were coded by a second independent observer, blind to the treatment each dog received on each day, to assess inter-rater reliability. Total duration of administration was also measured. Thirty minutes after OT or saline administration, dogs and owners participated in a modified attachment test, the Secure Base Test (modified from Harlow, 1958). This time period was chosen as previous work has shown that effects of OT can be seen after this time period (Woolley et al., 2014).

**Table 2:** Stress-related behaviors (Adapted from Deldalle and Gaunet, 2014).

**Behavior**	**Description**
Lip licking (frequency)	Dog licks lips
Yawning (frequency)	Dog opens mouth and yawns
Shivering (frequency)	Dog trembles
Whining (frequency)	Dog makes high pitched noise
Head shaking (frequency)	Dog moves head from side to side

**Figure 1:**
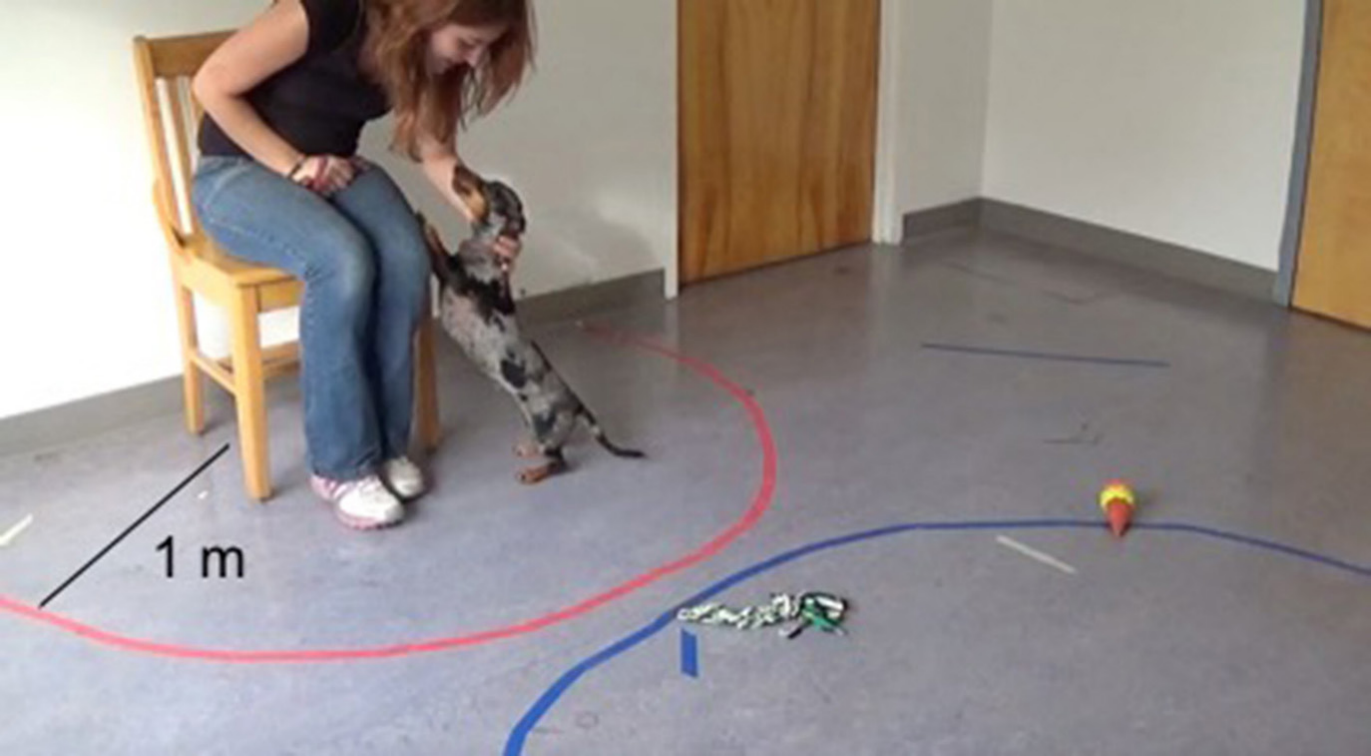
Layou of testing room for SBT. Written consent was obtained from this participant for the inclusion of this image in the manuscript.

The Secure Base Test occurred in a second unfamiliar room ~3.6 m by 4.2 m in size. The room contained a chair with a semi-circle 1 m in radius taped around the chair. Three dog toys were placed on the testing room floor during each testing session (Figure 1). Testing consisted of three phases, each lasting 2 min. Table 3 summarizes instructions that owners received for each phase of the attachment test. Each phase lasted for 2 min and immediately followed the phase preceding it, and the test began immediately after the dog and owner entered the testing room. In phase one (Baseline), the owner received instructions to sit neutrally in a chair in an unfamiliar testing room. Owners were able to reciprocate affection if the dog approached and entered the circle by petting the dog twice without restraining it by the collar if the dog approached (i.e., placed at least two paws or half of a body length inside the circle) or initiated contact. Dogs were able to freely explore the room. In phase two (Alone), the owner exited the testing room so that the dog was left alone. In phase three (Return), the owner re-entered the testing room. Owners were asked to sit neutrally, as in baseline, during this phase and were able to reciprocate affection by petting the dog twice without restraining it by the collar if the dog approached or initiated contact. Videos of the Alone phase were coded for search and separation anxiety behaviors (Table 4; McCrave, 1991; Overall et al., 2001; Storengen et al., 2014). Videos of the Baseline and Return phases were coded for attachment related behaviors (Table 5). An independent observer, blind to the treatment each dog received on each day, coded all videos. A second independent observer, also blind to condition, recoded 30% of the videos to ensure inter-rater-reliability.

**Table 3:** Owner instructions for attachment test.

**Phase**	**Description**
Phase 1 (Baseline) 2 min	Owners were instructed to sit in chair in testing room and pet the dog twice each time it entered the circle.
Phase 2 (Alone) 2 min	Owner and experimenter exited the room and the dog was left alone.
Phase 3 (Return) 2 min	Owner and experimenter quietly re-entered the room without greeting the dog. Owners were instructed to sit in chair in testing room and pet the dog twice each time it entered the circle. (Identical to baseline.)

**Table 4:** Alone phase focal behaviors.

**Behavior**	**Description**
Vocalizing (frequency)	Whining or barking
Touching or scratching at testing room door (frequency and duration)	Using any part of body to make contact with door
Elimination (frequency)	Urinating or defecating
Destruction (duration)	Destroying/chewing non-toy objects in testing room
Excessive motor activity (duration)	Pacing or other repetitive movements
Hypersalivation (frequency)	Excessive drooling or salivation
Exploring (duration)	Walking around room
Looking at door (frequency and duration)	Gazing in direction of door without making contact with door
Playing (duration)	Picking up/making contact with toys

**Table 5:** Attachment behaviors.

**Behavior**	**Description**
Inside circle (proximity seeking) (duration)	Laying, sitting or standing inside of the circle taped around the owner's chair
Outside circle (duration)	Laying, sitting or standing outside of the circle taped around the owner's chair
Exploring (duration)	Moving around the room or walking in a non-repetitive manner (i.e., not pacing)
Contact with owner (frequency and duration)	Physical contact with owner (or owner making contact with dog) with any part of body
Playing (duration)	Picking up/making contact with toys
Avoiding (duration)	Sitting, standing or laying out of reach outside circle

While duration-based measures are commonly used to evaluate the effects of OT administration on behavior, they may not be the most sensitive or reliable method for detecting the secure base effect or attachment style (Schöberl et al., 2016). Therefore, we also coded the videos using a holistic scoring approach specifically designed to assess attachment style categories (Schöberl et al., 2016) using the definitions in Table 6. For this method, all videos were double coded by two attachment style experts to ensure reliability. Both coders independently watched the Return phase of each video for each dog, and categorized the dogs according to the definitions in Table 6, allowing for an inter-rater reliability score. The two coders were then asked to come to a consensus as described by Schöberl et al. (2016) on all videos by jointly viewing videos where initial disagreement occurred and mutually deciding on an attachment style classification. Dogs where consensus could not be reached were labeled as unclassifiable (Schöberl et al., 2016).

**Table 6:** Attachment style definitions (adapted directly from Schöberl et al., 2016).

**Attachment Style**	**Definition**
Secure	Dog approaches owner promptly at reunion and follows, makes physical contact or signals for contact, seeks and is comfortable with contact. Little or no gaze aversion or proximity avoidance. Little or no resistance to contact or interaction.
Insecure avoidant	Dog shows little tendency to approach, to seek contact, or to follow. Dog turns or looks away during reunion. Dog shows lack of response to invitations to approach or interact for 30 s or more. Dog explores the room and objects during pre-separation and post-separation. There is little active search for owner.
Insecure ambivalent	On reunion, they mixed persistent distress with efforts to maintain physical contact and/or physically intrusive behavior directed toward the owner. These dyads were characterized by a degree of conflict regarding physical contact or play activities. For example, the dog wished to maintain contact and was uncooperative with the owner's attempt to encourage play or exploration, or the owner maintained firm physical contact which the dog merely passively tolerated. (Dogs who the judges agreed seemed essentially secure but with ambivalent tendencies, were included in the secure group).
Insecure Disorganized	Evidence of strong approach avoidance conflict or fear on reunion, for example, circling owner, hiding from sight, rapidly dashing away on reunion, “aimless” wandering around the room, shying away from contact, or proximity. “Dissociation” may be observed, that is, staring into space without apparent cause; still or frozen posture for at least 20 s (in the non-resting, non-sleeping dog).
Unclassifiable	Dogs showed ambiguous evidence of disorganization or other disturbance, for example, “depressed”-a marked lack of enthusiasm in a dog that otherwise seemed secure or showed other behavior suggesting a neurologic or compulsive disorder. Classifiers were unable to reach consensus on group placement for dogs from this classification category. Unclassifiable dogs were excluded from further analysis on dog attachment.

*Only descriptions pertaining to the return phase were used*.

### Statistical methods

All statistical analyses were conducted using RStudio. The frequency of each of the following stress-related behaviors was recorded during OT and saline administration for each testing session: lip licking, head shaking, shivering, whining, and yawning. The total amount of time that administration took was also recorded for each session. No instances of yawning were observed during administration for any dog in this study, so yawning was excluded from the analysis. The stress-related behaviors were coded as being mutually exclusive, and were summed to create an overall score of stress during administration. For analysis, data from each dog's first testing session was used so that for half of the dogs (*n* = 20), administration was performed with an MAD, and for half of the dogs (*n* = 20), administration was performed using a nasal spray bottle. Order of solution presentation (OT or saline) was counterbalanced equally between groups as well. Inter-rater reliability for duration of administration was 79.2 and 75% for the overall stress-related behavior score. The distribution of data for duration scores was heavily right skewed, therefore a log transformation of duration was used in the analysis which aided in the normalization of the data for analysis. Following this transformation the average duration of administration using the MAD vs. the nasal spray bottle method was compared using a *t*-test, as was the average number of stress-related behaviors for each administration type. Fishers Exact tests were used to compare the number of dogs with secure and insecure attachment styles for the MAD vs. the nasal spray bottle method for both saline and OT conditions separately. A power analysis was conducted during the procedural design phase of this study as recommended in the statistics literature (Das et al., 2016). It was determined that 20 subjects would give us 90% power to detect a large effect size, with a two-tailed alpha of 0.05, therefore a sample size of 20 subjects per administration type was utilized.

The variables measured for the baseline and return phases of testing include proportion of time spent engaging in the following activities: avoiding, exploring, inside the circle (a measure of proximity seeking), playing and contact with the owner, as well as the latency to enter the circle and the latency to make contact with the owner, and the number of times per session each dog made contact with its owner. Inter-rater reliability was high for all behaviors coded in the baseline and return phases: Baseline phase: mean 91% agreement, range 79–96% agreement across individual behaviors; Return phase: mean 86% agreement, range 75–92% agreement across individual behaviors. Histograms were used to assess normality. A Shapiro-Wilk test was used to assess normality of residuals for both treatment type (the within-subjects variable) and sex (the between-subjects variable) for each behavioral measure of interest within the Return phase. The assumption of normality was violated for the proportion of time dogs spent avoiding, exploring, in proximity to their owners, playing, in contact with their owners, the latency to make contact with their owners and the latency to seek proximity to their owners. The normality assumption was not violated for the frequency at which dogs made contact with their owners. A paired *t*-test was used to compare proportion of time spent inside the circle when dogs were given OT vs. when they were given saline.

For the Alone phase, we were interested in the relative frequency of behaviors associated with the presence or absence of separation distress as identified in canine attachment (Schöberl et al., 2016) and separation anxiety (McCrave, 1991; Overall et al., 2001; Storengen et al., 2014) literature. The proportion of time the dog spent engaging in the following activities was recorded: being out of sight, looking at the door, playing, and touching the door. The number of times each dog engaged in hypersalivation, elimination, repetitive movement, vocalizing, and destruction, touching the door, and looking at the door during the alone phase was measured as well. No instances of hypersalivation, elimination, repetitive movement, or destruction were observed during the alone phase of the attachment test, so these behaviors were excluded from all analyses of the alone phase. While duration of looking at the door (38% agreement), frequency of looking at the door (63% agreement) and number of vocalizations (38% agreement) produced during the alone phase had moderate reliability scores, all other reliability measures indicated strong agreement: mean 93%, range 75–100% agreement). Histograms were used to assess normality. The Shapiro-Wilk test was used to assess normality of residuals for both treatment type (the within-subjects variable) and sex (the between-subjects variable) for each behavioral measure of interest within the Alone phase. The assumption of normality was violated for the proportion of time dogs spent out of sight, exploring, playing, and touching the door, the frequency at which dogs touched the door and the frequency at which dogs vocalized for both treatment and sex. However, the mixed design ANOVA is generally considered robust to violations of normality, and therefore was chosen to allow for the targeted evaluation of interaction effects. The normality assumption was not violated for the frequency at which dogs looked at the door or the proportion of time dogs spent looking at the door for either treatment type or sex. Within-subject comparisons were analyzed using a paired *t*-test to determine whether any differences were present when dogs were given OT vs. when they were given saline with respect to the variables measured during the alone phase of the attachment test.

To investigate the interaction between treatment and subject sex in the alone and return phases, we also compared the behavior of males and females when given OT or saline with a 2 × 2 Mixed Design ANOVA. This is an important consideration as studies have demonstrated that male and female dogs/humans can show different behavioral trends after OT administration (Nagasawa et al., 2015; Oliva et al., 2015).

Inter-rater reliability for holistic coding, when two independent coders categorized dogs according to the definitions outlined in Table 6, was 77.5%. In order to ensure that all dogs were reliably categorized according to the appropriate attachment styles, the same two coders re-watched the videos for the dogs for which they did not independently agree and mutually decided on an attachment style (Waters, 1978; Schöberl et al., 2016). Videos for three dogs were scored as unclassifiable and were dropped from analysis (resulting in six total testing sessions being dropped from this portion of the analysis). A Fishers Exact test to compare the number of dogs categorized as having a secure attachment (demonstrating the secure base effect) after administration of OT vs. saline.

All tests were two tailed and had an alpha level of 0.05 unless otherwise specified. *Post-hoc* comparisons were made using *t*-tests with a Tukey-Kramer adjustment for all pairwise comparisons.

## Results

### Administration

Results indicated that administration in the MAD group (Mean duration = 18.25 s) was shorter compared to the nasal spray bottle group (Mean duration = 32.75 s), this difference was not statistically significant [*t*_(38)_ = −1.8019, *p* = 0.08]. No significant differences were observed with respect to overall stress-related behavior between administration types [*t*_(27.679)_ = −1.2303, *p* = 0.23]. No significant differences were found with respect to attachment style based on administration type when either the saline (*p* = 1.00) or the OT solutions (*p* = 1.00) were administered. As no significant differences were found on these measures based on administration type, data from both administrative methods was pooled for the remaining analyses. However, since sample size is relatively small (*n* = 20 per group) when grouped according to administration type, we have also included analyses (using a Mixed Design ANOVA) for MAD and nasal spray bottle groups separately.

### Duration and frequency based measures: baseline phase

Dogs that received saline spent significantly more time inside the circle compared to dogs that received OT [*t*_(39)_ = 2.11, *p* = 0.04]. On average, dogs spent 6.5% more time in proximity to their owners when saline was administered compared to when OT was administered (see Table 7). No other measures in the Baseline phase differed significantly when dogs received OT compared to when they received saline. While we predicted that the proportion of time dogs spent in contact with their owners would significantly differ when they were given OT vs. saline-based on the proximity and contact seeking effects previously reported (Romero et al., 2014)—this was not observed in the current study [*t*_(39)_ = 0.35, *p* = 0.73]. Additionally, a binomial test was used to compare the number of dogs that showed an increase in proximity seeking when given OT compared to saline. A total of 17 dogs out of 40 showed an increase in proximity seeking after OT administration when compared to saline (*p* = 0.43). Of these dogs, 6 dogs belonged to the MAD group and 11 dogs belonged to the nasal spray bottle group.

**Table 7:** Effect sizes for behaviors of interest for overall comparisons with pooled data.

	**OT**		**Saline**				**95% CI**		
**Variable**	***M***	***SD***	***M***	***SD***	***t*_(39)_**	***p***	***LL***	***UL***	**Cohen's *d***
Baseline-Inside circle (proximity seeking, duration)	0.36	0.26	0.42	0.25	2.11	0.04	0.002	0.13	0.33
Alone- Vocalizing (frequency)	32.45	27.81	27.10	25.65	−1.87	0.07	−11.15	0.45	−0.30

For comparisons between administration type, a significant interaction was found between method of administration and treatment for avoidance behavior [*F*_(1, 1)_ = 4.67, *p* = 0.04]. A trend was found with respect to saline administration, as, on average, dogs in the nasal spray bottle group spent 8.3% more time exhibiting avoidance behavior than dogs in the MAD group [*t*_(31.95)_ = −1.90, *p* = 0.07], see Table 8. Within-subjects comparisons for the MAD group revealed that there was a trend of dogs spending an average of 6.1% more time exhibiting avoidance behavior when OT was administered compared to when saline was administered [*t*_(19)_ = 1.93, *p* = 0.07]. For a summary, see Table 9. Averages for time spent displaying avoidance behavior for dogs in the MAD group include 9.62% when OT was administered and 3.62% when saline was administered. Dogs in the nasal spray bottle group spent an average of 8.20% of the session displaying avoidance behavior when OT was administered and 11.92% of the session when saline was administered. *Post-hoc* comparisons did not reveal any other trends or significant differences. With respect to proximity seeking, no significant interactions between method of administration and treatment were found. On average, dogs in the MAD group spent 36.98% of the session in proximity to their owners when OT was administered and 46.09% of the session in proximity to their owners when saline was administered. Dogs in the nasal spray bottle group, on average, spent 35.60% of time in proximity to their owners when OT was administered and 38.54% of the session in proximity to their owners when saline was administered. No significant interactions between method of administration and treatment were found for any other behaviors measured in baseline.

**Table 8:** Effect sizes for behaviors of interest for dogs by administration type (i.e., with separate analyses for MAD and nasal spray bottle administration types) in the saline condition.

	**MAD**		**Nasal spray bottle**				**95% CI**		
**Variable**	***M***	***SD***	***M***	***SD***	***t*_(31.95)_**	***p***	***LL***	***UL***	**Cohen's *d***
Baseline-Avoiding (duration)	0.04	0.10	0.12	0.17	−1.90	0.07	−0.17	0.006	−0.57

**Table 9:** Effect sizes for behaviors of interest for dogs by solution type in the MAD condition only.

	**OT**		**Saline**				**95% CI**		
**Variable**	***M***	***SD***	***M***	***SD***	***t*_(19)_**	***p***	***LL***	***UL***	**Cohen's *d***
Baseline-Avoiding (duration)	0.096	0.19	0.04	0.10	1.93	0.07	−0.005	0.13	0.43

### Duration and frequency based measures: alone phase

No statistically significant differences were observed with respect to any of the variables of interest. However, there was a trend of dogs vocalizing more during the alone condition when OT was administered compared to when saline was administered [*t*_(39)_ = −1.87, *p* = 0.07]. On average, dogs vocalized 5.4 times more when OT was administered than when saline was administered. Overall, no significant differences or trends were found according to type of administration device used for any of the behaviors coded in the alone phase. Dogs assigned to the MAD administration group vocalized an average of 31.2 times during the alone phase when OT was administered and an average of 26.15 times when saline was administered. For the nasal spray bottle group, dogs vocalized an average of 33.7 times during the alone phase when OT was administered and an average of 28.05 times when saline was administered.

Only moderate effects of treatment, sex or treatment by sex interaction were found with respect to the behavioral variables of interest. A trend was seen with respect to treatment on the frequency at which dogs vocalized [*F*_(1, 1)_ = 3.40, *p* = 0.07]. There was a trend of an interaction between treatment and sex for the frequency at which dogs looked at the door [*F*_(1, 1)_ = 3.7, *p* = 0.06]. When saline was administered, there was a trend in which males tended to look at the door with a greater frequency than did females, *t*_(30.13)_ = −2.00, *p* = 0.05, with males looking at the door 2.11 more times than females. In the saline condition, females spent significantly more time touching the door than did males [*t*_(21.26)_ = 2.28, *p* = 0.03]. On average, females spent 9.5% more time in contact with the door than did males (see Table 10).

**Table 10:** Effect sizes for behaviors of interest for comparisons of males and females with pooled data.

	**Females**		**Males**				**95% CI**		
**Variable**	***M***	***SD***	***M***	***SD***	***t*(df)**	***p***	***LL***	***UL***	**Cohen's *d***
Alone- Looking at door (duration)-saline condition	7.27	2.71	9.39	3.76	−2.00	0.05	−4.28	0.04	−0.65
Alone- Contact with door (duration)-saline condition	0.10	0.19	0.007	0.14	2.28	0.03	0.008	0.18	0.56
Return- Contact with owner (latency)-saline condition	2.86	3.01	16.67	37.44	1.75	0.09	−29.99	2.37	0.52
Return-Playing (duration)-OT condition	0.15	0.21	0.31	0.35	−1.75	0.09	−0.35	0.03	−0.55

### Duration and frequency based measures: return phase

No significant differences were found with respect to the variables of interest in the return condition when within-subject comparisons were made. No significant differences or trends were found for any of the behaviors observed in the return phase with respect to type of administration device used. Of special interest was the fact that there was not a statistically significant difference with respect to the proportion of time dogs spent in proximity to their owners when treated with OT vs. saline [paired *t*-test, *t*_(39)_ = 0.63, *p* = 0.53]. On average, dogs in the MAD group spent 57.02% of the return session seeking proximity when OT was administered, and 59.0% of time seeking proximity when saline was administered. Dogs in the nasal spray bottle group spent an average of 52.36% of the session seeking proximity when OT was administered and 56.35% of the session seeking proximity when saline was administered. There was also not a significant difference in the proportion of time dogs spent in contact with their owners when treated with OT vs. saline [paired *t*-test, *t*_(39)_ = 0.59, *p* = 0.55]. Dogs assigned to the MAD group spent an average of 19.36% of the session in contact with owners when OT was administered and an average of 19.05% of time in contact with their owners when saline was administered. Dogs assigned to receive administration via a nasal spray bottle spent an average of 16.83% of time in contact with their owners after OT administration and an average of 20.86% of the session in contact with owners after saline administration.

To investigate a possible interaction between treatment and sex, we compared the behavior of males and females when given OT or saline with a 2 × 2 Mixed Design ANOVA. While no statistically significant differences were found, there was a trend of an effect of sex on latency to make contact with their owners [*F*_(1, 1)_ = 2.90, *p* = 0.09]. A trend was present in which females had a shorter latency to engage in contact with their owners than males in saline condition [*t*_(38)_ = 1.75, *p* = 0.09], with females making contact with their owners an average of 13.8 s faster than males. A trend was seen with respect to playing, as there was a tendency for males to spend a greater proportion of time engaging in play than females after OT administration [*t*_(26.28)_ = −1.75, *p* = 0.09]. On average, males spent 16.3% more time engaging in play compared to females after OT administration. See Table 10 for a summary of effect sizes for results of interactions between treatment and sex.

### Evaluation of the secure base effect and attachment-style

Attachment style categorizations for each dog for both OT and saline conditions can be found in Table 1. For the OT condition, 31 dogs were scored as secure, 3 dogs were scored as insecure avoidant, and three dogs were scored as insecure ambivalent. For the saline condition, 32 dogs were scored as secure, two dogs were scored as insecure avoidant, two dogs were scored as insecure ambivalent, and one dog was scored as insecure disorganized. The Fisher's exact test comparing the number of dogs with secure and insecure attachments for both the OT and saline conditions was not significant (*p* = 1.00), indicating that OT did not impact the attachment styles of dogs in the present study. Only one dog was scored as insecurely attached in the OT condition but securely attached in the saline condition. The remaining dogs who were scored as insecure, were scored as being insecurely attached in both phases (although the type of insecure attachment did vary for a few individuals). Overall, attachment style category changes were only observed for three dogs: one dog was classified as insecure avoidant when OT was administered, but was categorized as insecure disorganized when saline was administered, one dog was classified as insecure avoidant when OT was administered but scored as secure when saline was administered, one dog was classified as insecure ambivalent when OT was administered, but was classified as insecure avoidant when saline was administered.

## Discussion

**Figure 2:**
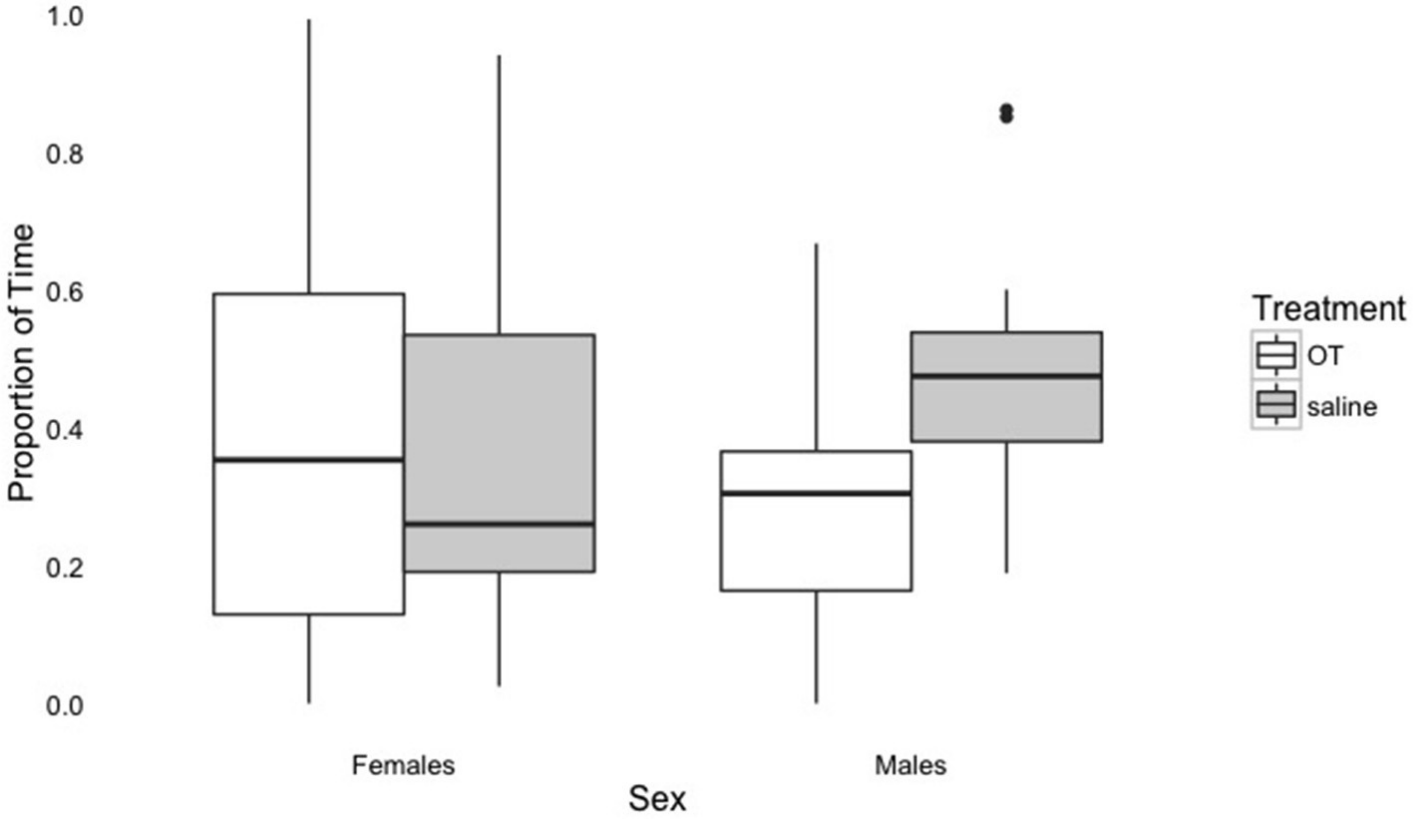
Proportion of time spent within 1 m of the owner in the Baseline phase by treatment and sex. The dark line indicates the median, the box indicates the interquartile range, or the middle portion of the data, the upper bar indicates scores above the middle 50%, the lower bar indicates scores below the middle 50%, and outliers are greater than the upper bar by more than 1.5 times the interquartile range.

The results of the baseline phase were unexpected, as previous literature has shown that OT results in increased affiliative behavior and proximity seeking (Romero et al., 2014; Nagasawa et al., 2015). In contrast, we found that dogs sought proximity for longer durations after the saline administration, not OT administration. One possible explanation for this result may have to do with the relationship between the effects of OT and sex. For example, some research has suggested that female dogs may be more sensitive to the prosocial effects of OT and males may exhibit increased vigilance after administration of OT, particularly if OT binds to receptors for vasopressin, a structurally similar molecule (Nagasawa et al., 2015). As Figure 2 shows, male dogs tended to spend less time in proximity to their owner (within the 1 m circle) when given OT, while females do not differ with respect to the proportion of time spent in owner proximity when given OT or saline. Thus, it is possible that OT resulted in increased vigilance in males that led them to spend less time in proximity to their owners during baseline driving this effect. Evidence from studies with both humans and prairie voles indicates that vasopressin has sexually dimorphic effects, and it is associated with defensive behaviors (for a review, see Carter, 2014). Thus, if OT binds to vasopressin receptors, it could lead to an increase in these behaviors, particularly for males. In humans, vasopressin has also been shown to increase defensive behaviors in an adaptive manner (Heinrichs and Domes, 2008) and plays a role in bonding and aggressive behavior by increasing encoding of positive and negative social cues (Guastella et al., 2010).

The effects of OT on the behavior of dogs during an Alone condition where their owner leaves them alone in an unfamiliar room, had never previously been evaluated. This condition has important implications for the potential mechanisms underlying behavioral responses associated with OT. We hypothesized that if administration of OT resulted in greater attachment security, that when given OT, dogs would engage in fewer stress-related behaviors during a brief separation period from their owners compared to the saline control condition. The results did not support this hypothesis. Instead, there was a trend of dogs vocalizing more frequently when they had received OT compared to when they received saline. As vocalizing may indicate stress, it is possible that administration of OT may increase stress when dogs experience a short separation period from their owners. Based on the findings of prior studies demonstrating proximity seeking behavior after OT administration (Romero et al., 2014), increased vocalizations or stress when left alone could be due to the disruption of proximity seeking behavior at a time when motivation to engage in this behavior is especially high. However, it should be noted that this effect was minimal, at least among the pet dogs tested in this study, and that in the current study we did not find a significant increase in proximity seeking behavior by dogs toward their owners during baseline. There was also a trend of males spending a greater proportion of time looking at the door of the testing room in their owner's absence in the saline condition, but this effect was not seen with OT. This may suggest that there are sex differences that predict owner-directed search-related behaviors in the absence of their owners, and OT may decrease these differences.

As two different methods of administration were tested in this study, we conducted additional analyses without pooling data for both groups. Overall, the only instance an interaction effect between type of administration and behavior was for avoidance behavior in the baseline phase. However, it should be noted that only 19 of 40 dogs exhibited any avoidance behavior in baseline when OT was administered, and only 19 of 40 dogs displayed avoidance behavior when saline was administered. In addition, it should be noted that although differences in duration of administration and overall stress experienced during administration were not statistically significant, sample size was relatively small (*n* = 20 per administration type). Thus, it is possible that different methods of nasal administration could lead to different outcomes in behavioral tests, although limited evidence of this was found in the present study.

Additionally, we hypothesized that OT administration would result in an increase in proximity seeking and/or behaviors associated with secure attachment when dogs were reunited with their owners after a short separation period. No such effect was found. A trend toward a greater proportion of time spent engaging in play was identified for males, but not females, when OT was administered. Play is often thought to be an indicator of welfare (Held and Špinka, 2011) and is sometimes used as a measure of the secure base effect during the reunion phase of attachment tests (Schöberl et al., 2016), so a trend toward an increase in time spent engaging in play could suggest that OT may have some impacts on welfare. However, the relationship between the increase in play behavior and OT administration for males was not statistically significant and other methods, such as direct human interaction and petting (Mehrkam et al., 2014), have been found to have a more robust and immediate effect on rate of play.

Overall, while some significant differences were found between OT and saline conditions, and between male and female responses to OT administration, such differences are often modest in both this and other studies. For example, several studies have found fairly small effect sizes of nasally administered OT in dogs including in relation to dogs' ability to use pointing gestures to find hidden food in an object-choice task (Oliva et al., 2015) responses to the threatening approach of owners (Hernádi et al., 2015), and relatively small changes in affiliation rate with familiar humans and conspecifics (Romero et al., 2014). While the temptation might be to increase sample size, this raises an important issue for future research. Studies evaluating intranasal administration of OT are often concerned with the effect of treatment on the behavior of individual animals, especially in cases where OT administration might be recommended as a behavior modification tool or aid (Thielke and Udell, 2015). Therefore, increased sample size for studies of this type might actually be problematic, as a larger body of averaged data could mask the relative weakness of behavior change that might be expected for a single dog. In contrast, other treatments have shown a greater behavioral effect with similar sample sizes. For instance, one study comparing the efficacy of a dog appeasing pheromone to clomipramine (an antidepressant medication) for the treatment of separation anxiety in 57 dogs measured improvement on several different behaviors before and after either treatment intervention (Gaultier et al., 2005). The smallest improvement was seen in 65% of dogs vocalized less or did not vocalize at all in the absence of their owners after treatment. Therefore, an improvement in separation anxiety symptoms was seen for the majority of dogs in the study, regardless of treatment type. Therefore, future research should attempt to further explore predictive variables that could explain the different degrees of behavioral change reported across studies investigating the effects of OT administration, at both the individual and group level, such as sex differences, OT dosage or administration methods, and not simply increase sample size. Conversely, some researchers and clinicians may find moderate effect sizes informative for some applications, therefore providing a diversity of information on the effects of OT administration with different sample sizes and effect sizes will likely be important for future directions.

The current study was also conducted with pet dogs without known anxiety disorders, and although some effects of OT have been found on the social behavior of this population in the past, more substantial effects may still be found in specific social populations, for example in dogs with separation anxiety. Furthermore, many pet dogs in this and other (e.g., Schöberl et al., 2016) attachment studies have demonstrated a secure attachment to their owners. For this reason, pro-social changes associated with OT administration may be less detectable in the general population, but might be more salient for dogs who initially display insecure attachments to their owners. It should also be noted that the present study did not include measuring dogs' plasma OT levels after OT administration, therefore we cannot rule out the possibility that OT levels decreased during the waiting period for the SBT. While a waiting period between OT administration and behavioral testing is considered standard practice the duration of the waiting period itself varies across studies. Although Romero et al. (2014) suggests an optimal waiting period of about 15 min, one study used 40 min waiting periods (Hernádi et al., 2015), while another employed a 45 min waiting period (Oliva et al., 2015). Variation in waiting period is also found in studies with other species, including humans, for example one study used a 30 min waiting period (Woolley et al., 2014), while another used a waiting period that ranged from 45 to 90 min (Guastella et al., 2009). More research is needed in both of these areas to determine if specific populations or methods may lead to greater or more consistent affects of OT on behavior than others.

In addition, while some studies involving nasal OT administration in dogs have also used double-blind methodologies (Hernádi et al., 2015; Kis et al., 2015; Oliva et al., 2015, 2016; Kovács et al., 2016) as was the case in the current study, other studies have used a single-blind procedure in which either the owner or coders were not aware of which treatment was given at each session or to each subject, but where the experimenters conducting the study may have known which treatment was administered at each session (Romero et al., 2014; Nagasawa et al., 2015). Future research should consider to what degree experimenter knowledge or bias could influence the behavior of dogs or their owners in studies of this type.

Finally, it is worth noting that these findings are similar to findings from research with human subjects, where OT has been shown to have nuanced effects-increasing prosocial behavior in some contexts, while yielding negative results or leading to antisocial behavior in other contexts. For a review, see (Bartz, 2016). As a result, applications of OT in applied contexts, may be limited or minimally require further investigation targeting subpopulations experiencing specific behavior problems or disorders, as the effect may be larger in these populations compared to the general population.

## Author contributions

LT, GR, SS, and MU designed the study. LT collected the data. LT and MU performed data analyses and interpreted the data. LT and MU wrote the first draft of the manuscript. LT, GR, SS, and MU revised the manuscript.

### Conflict of interest statement

The authors declare that the research was conducted in the absence of any commercial or financial relationships that could be construed as a potential conflict of interest.
